# Peg-IFNα combined with hepatitis B vaccination contributes to HBsAg seroconversion and improved immune function

**DOI:** 10.1186/s12985-024-02344-8

**Published:** 2024-03-30

**Authors:** Yisi Liu, Shan Ren, Lina Ma, Xiao Lin, Junfeng Lu, Zhenhuan Cao, Sujun Zheng, Zhongjie Hu, Xiaoxue Xu, Xinyue Chen

**Affiliations:** 1 First Department of Liver Disease Center, Beijing Youan Hospital, Capital Medical University, No.8, Xi Tou Tiao, Youanmen wai, Beijing, 100069 China; 2 Third Department of Liver Disease Center, Beijing Youan Hospital, Capital Medical University, No.8, Xi Tou Tiao, Youanmen wai, Beijing, 100069 China; 3 Department of Core Facility Center, Capital Medical University, No.8, Xi Tou Tiao, Youanmen wai, Beijing, 100069 China

**Keywords:** Hepatitis B vaccine, HBsAg seroconversion, Interferon therapy, Humoral immunity, Functional cure

## Abstract

**Purpose:**

The purpose of this study was to investigate immunological variations between a group that received the hepatitis B vaccine and a non-vaccine group. We focused on a cohort that achieved HBsAg seroclearance after Peg-IFNα treatment of CHB.

**Methods:**

We enrolled twenty-eight individuals who achieved HBsAg seroclearance after Peg-IFNα treatment. They were divided into two groups: a vaccine group (*n* = 14) and a non-vaccine group (*n* = 14). We assessed lymphocyte subpopulations, B cell- and T cell-surface costimulatory/inhibitory factors, cytokines and immunoglobulin levels were detected at different time points to explore immune-function differences between both groups.

**Results:**

The seroconversion rate in the vaccine group at 24 weeks post-vaccination was 100%, which was significantly higher (*p* = 0.006) than that of the non-vaccine group (50%). Additionally, more individuals in the vaccine group exhibited anti-HBs levels exceeding 100 IUs/L and 300 IUs/L compared to the non-vaccine group (*p* < 0.05). The vaccine group demonstrated significantly increase total B cells and class-switched B cells at 24 weeks and plasma cells, CD80^+^B cells, Tfh cells, and ICOS^+^Tfh cell at 12 weeks, compared with baseline levels (*p* < 0.05). Conversely, Bregs (CD24^+^CD27^+^ and CD24^+^CD38^high^) decreased significantly at 24 weeks (*p* < 0.05). None of the above changes were statistically significance in the non-vaccine group (*p* > 0.05). Total IgG increased significantly in the vaccine group, and IL-2, IL-5, and IL-6 concentrations increased significantly at week 24 (*p* < 0.05). Differences in various types of cytokines and immunoglobulins in the plasma of the non-vaccine group were not significant (*p* > 0.05). Anti-HBs titers positively correlated with Th1/Th2 cells at 24 weeks (*r* = 0.448 and 0.458, respectively, *p* = 0.022 and 0.019, respectively), and negatively with CD24^+^CD38^high^Breg cells (*r* = -0.402, *p* = 0.042).

**Conclusions:**

After achieving HBsAg seroclearance through Peg-IFNα treatment for CHB, administering the hepatitis B vaccine significantly increased anti-HBs-seroconversion rates and antibody levels. We also observed significant immunological differences between the vaccine and non-vaccine groups. Specifically, the vaccine group exhibited significant increases in B cells, plasma cells, and Tfh cells, while Breg levels was significantly lower. These immunological changes are likely conducive to the production of anti-HBs antibodies. However, in the non-vaccine group, the observed changes were not significantlly significant.

**Supplementary Information:**

The online version contains supplementary material available at 10.1186/s12985-024-02344-8.

## Background

Hepatitis B virus (HBV) infection is a major cause of cirrhosis and hepatocellular carcinoma (HCC), posing a serious global public health problem [1]. Previous findings showed that hepatitis B surface antigen (HBsAg) seroclearance resulted in improved liver histology and a reduced incidence of HCC [2, 3]. Consequently, HBsAg seroclearance is considered a functional cure for chronic hepatitis B (CHB) infection with a desirable therapeutic endpoint and is recommended by guidelines for CHB prevention and treatment [4, 5]. In recent years, an increasing number of studies focusing on maintaining the HBsAg-seroclearance status after treatment have revealed that hepatitis B surface antibodies (anti-HBs) seroconversion and high antibody levels are correlate strongly with lower HBsAg-recurrence rates [6–8]. To mitigate HBsAg recurrence, researchers have administered hepatitis B vaccines to elevate anti-HBs levels in CHB patients with HBsAg seroclearance. For instance, in a study including 11 patients who achieved HBsAg seroclearance after receiving a single dose of hepatitis B vaccine, the anti-HBs seroconversion rate at the 9-month follow-up was 81.8% [9]. In another prospective study with 33 individuals who experienced recurrence after HBsAg seroclearance and who were re-treated with pegylated-interferon α (Peg-IFNα), all 18 patients who received hepatitis B vaccination were positive for anti-HBs, with only 26.7% of the 15 patients who did not receive hepatitis B vaccination developed antibodies [10]. These findings underscore the effectiveness of hepatits B vaccination in increasing anti-HBs levels among individuals with HBsAg seroclearance. However, the impact of hepatits B vaccination on immune functions remains less explored. Therefor, we investigated whether hepatitis B vaccination affects immune functions in CHB patients and assessed correlations between immune functions changes and anti-HBs production in a population that achieved HBsAg seroclearance following Peg-IFNα treatment.

## Methods

### Study population and design

The study population consisted of patients with CHB infection who visited Beijing Youan Hospital, Capital Medical University between November, 2018 and February, 2023 and achieved HBsAg seroclearance after Peg-IFNα treatment. The enrolled patients with HBsAg seroclearance met the following criteria: HBsAg < 0.05 IUs/mL, anti-HBs < 10 IUs/L, HBeAg-negative, HBV DNA below the lower limit of detection, and normal alanine transaminase (ALT) levels. These patients received Peg-IFNα consolidation therapy for 24 weeks after HBsAg seroclearance. The patients were divided into vaccinated and non-vaccinated groups according to whether they received hepatitis B vaccination after HBsAg seroclearance. All 14 patients in the vaccinated group were vaccinated with 20 µg of hepatitis B vaccine every 4 weeks for a total of six injections during the Peg-IFNα consolidation-therapy period, and none of the 14 patients in the non-vaccinated group received hepatitis B vaccination during consolidation therapy. Peripheral blood specimens were collected for clinical laboratory testing and immunological testing at the time of HBsAg seroclearance (baseline or 0 week), at 12 weeks, and 24 weeks in the vaccinated group, and at baseline and 24 weeks in the non-vaccinated group. Relevant adverse reactions were recorded in detail during the follow-up treatment period. This study was approved by the Ethics Committee of Beijing Youan Hospital (approval number [2018]050), and all patients provided written informed consent.

### Blood sample processing

Peripheral venous blood samples were transferred to ethylenediaminetetraacetic acid tubes (DB) and processed within 24 h. Briefly, the tube contents were placed in Ficoll–Paque density-gradient centrifuge tubes (GE Healthcare) and centrifuged at 2000 rpm for 10 min. Pasteur pipettes were used to transfer serum and peripheral blood mononuclear cells (PBMCs) to cryovials and stored at -80 °C for later use.

### Laboratory tests and methods

We detected HBV serological markers using the Elecsys-2010 system (Roche, Mannheim, Germany) with a lower limit of detection of 0.05 IU/mL for HBsAg and 2 IUs/L for anti-HBs. HBeAg ≥ 1 and anti-HBs ≥ 10 IUs/L were used as cut-offs for positive results. HBV DNA was measured using the COBAS TaqMan fluorescent quantitative polymerase chain reaction system (Roche) with a lower limit of detection of 20 IU/mL. Liver-function tests were performed using an OLYMPUS-AU5400 biochemical analyzer (Shinjuku, Japan), with a normal range of 9–40 U/L for ALT.

### Mass cytometry

Thawed PBMC were transferred to a 37 °C centrifuge tube containing RPMI-1640 medium and resuspended in phosphate buffered saline (PBS). PBMCs were washed with PBS on ice, stained with cisplatin (195-Pt, Fluidigm, USA), and cell viabilities were assessed. Purified antibodies were purchased from BioLegend and then conjugated with metals using the Maxpar® X8 Multimetal Labeling kit (Fluidigm) according to the manufacturer’s protocol. The list of antibodies and reporter isotopes are described in detail in Supplementary Table 1. Next, PBMCs were stained with cell-surface antibodies, and intracellular staining was performed using the Intercalator-Ir reagent (Fluidigm). Finally, the specimens were resuspended in deionized water containing 10% EQ Four Element beads (Fluidigm). A Helios mass cytometer (Fluidigm) was used for data acquisition.

### Cytokine and immunoglobulin (ig) detection with Luminex kits

Cytokine and Ig in plasma were analyzed by performing Luminex bead-based MILLIPLEX assays. MILLIPLEX panel kits were used to measure interleukin (IL)-2, IL-4, IL-5, IL-6, IL-10, IL-12, IL-17, IL-22, IFN-γ, tumor necrosis factor (TNF)-β, IgA, IgG1, IgG2, IgG3, IgG4, and IgM levels. We used the xPONENT software to read and quantify the data, which was generated using the FlexMAP3D (Luminex) platform.

### Statistical analysis

**Fig. 1:**
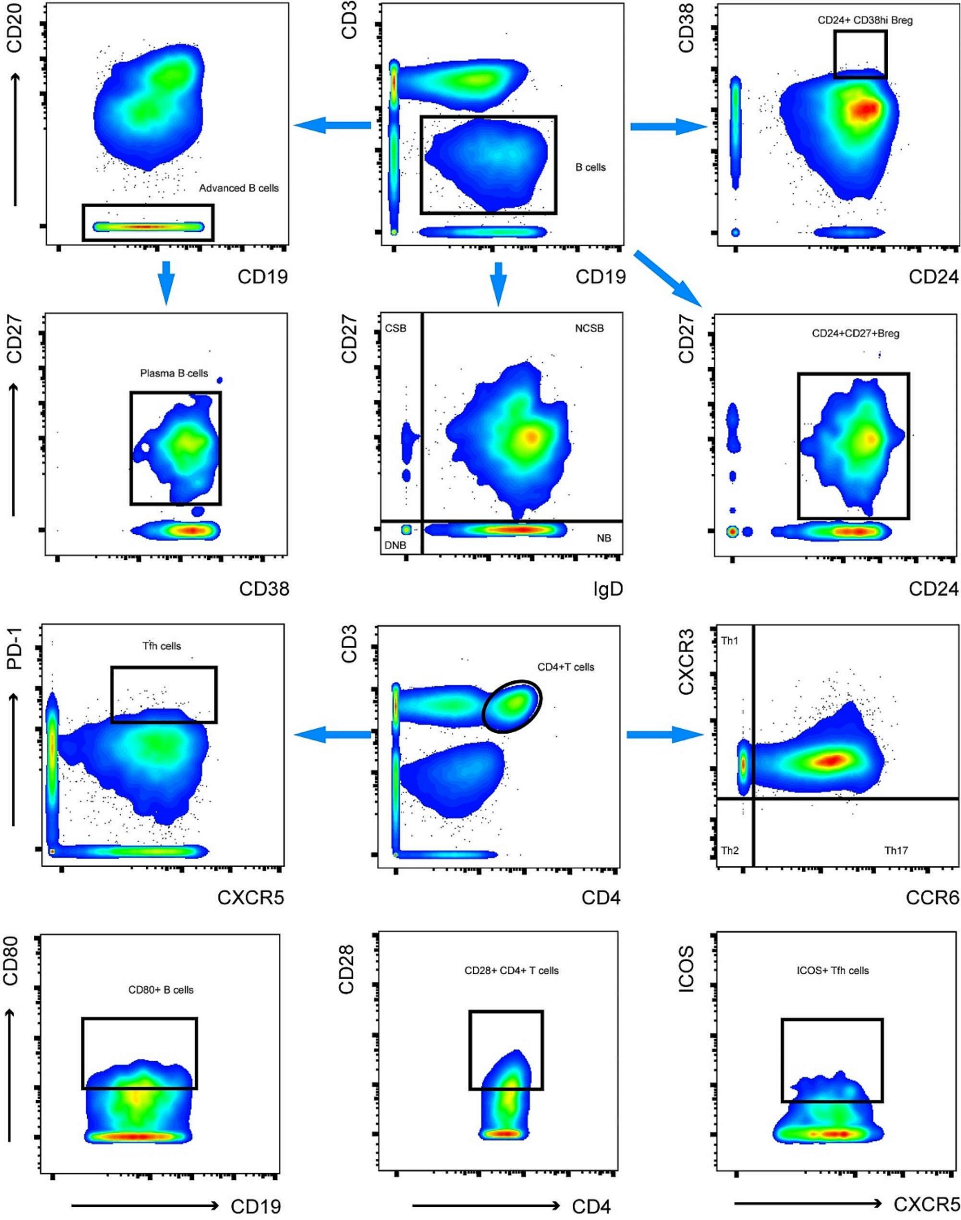
The gating strategy of B cells and CD4^**+**^T cells populations. CD19^+^cells were gated. Plasma cells were determined as CD19^+^ CD20^−^ CD38^+^ CD27^+^ cells. Class switch B cells (CSB: CD19^+^ IgD^−^ CD27^+^), Non class switch B cell (NCSB: CD19^+^ IgD^+^ CD27^+^), Double negative B cell (DNB: CD19^+^ IgD^−^ CD27^−^), Naive B cell (NB: CD19^+^ IgD^+^ CD27^−^) subsets were gated based on gated B cells. Two subsets of regulatory B cells (Breg) cells were determined as CD19^+^ CD24^+^ CD38^high^ and CD19^+^ CD24^+^ CD27^+^ cells. Then CD3^+^ CD4^+^ cells were gated. Follicular helper T cells (Tfh cells) were determined as CD3^+^CD4^+^ CXCR5^+^PD-1^high^ cells. Th1, Th2 and Th17 cells were identified as CXCR6^−^CXCR3^+^ (Th1), CXCR6^−^CXCR3^−^ (Th2) and CXCR6^+^CXCR3^−^ (Th17) within CD3^+^CD4^+^ group. CD80, CD86 and CD40 were gated based on gated CD19^+^cells. CD28 and CTLA4 were gated based on gated CD3^+^ CD4^+^ cells. ICOS and IL-21 were gated based on gated Tfh cells

Each mass cytometry sample was manually gated and exported from Cytobank. Mass cytometry results were analyzed using the FlowJo software. The gating strategy for the B cell and CD4^+^T cell subsets is shown in Fig. 1. Cytokine-expression levels were expressed as the percentage of positive cells to the total number of cells for each cell type. Data and image processing were performed using R and GraphPad Prism software. The independent samples *t*-test or paired *t*-test was used to compare groups with normally distributed measurements, and the Wilcoxon test was used compare groups with skewed measurements. Linear relationships between measurements were analyzed using Spearman’s correlation coefficient. A *p*-value of < 0.05 was considered to indicate a statistically significant difference.

## Results

### Patient demographics

In this study, there were two groups: the vaccinated group and the non-vaccinated group. Each group consisted of 14 individuals. Among these participants, there were 16 males and 12 females. The average age of all participants was 36.46 ± 10.55 years. At the beginning of the study (baseline), there were no significant differences between the two groups in terms of sex ratio, age, liver funcion, or cytokine levels (Table 1 and Supplementary Table 2).

**Table 1:** Comparison of baseline data and 24-week anti-HBs levels

	Vaccine group (*n* = 14)	Non-vaccine group (*n* = 14)	*p* value
Sex, Male, n (%) Female, n (%)	8 (57.1%) 6 (41.9%)	8 (57.1%) 6 (41.9%)	1
Age, years Range	35.64 ± 7.49 26–51	37.29 ± 13.19 27–55	0.688
ALT at baseline (U/L)	19.64 ± 5.94	16.64 ± 5.31	0.171
Anti-HBs positive conversion (≥ 10 IU/L) rate at week 24	100% (14)	50% (7)	0.006
Median of Anti-HBs at week 24, IU/L	409.3	8.97	0.007
Proportion of anti-HBs ≥ 100 IU/L at week 24, % (n)	64.3% (9)	21.4% (3)	0.022
Proportion of anti-HBs ≥ 300 IU/L at week 24, % (n)	57.1% (8)	14.3% (2)	0.018

ALT; alanine aminotransferase

### Comparison of antibody levels between the vaccinated and non-vaccinated groups

**Fig. 2:**
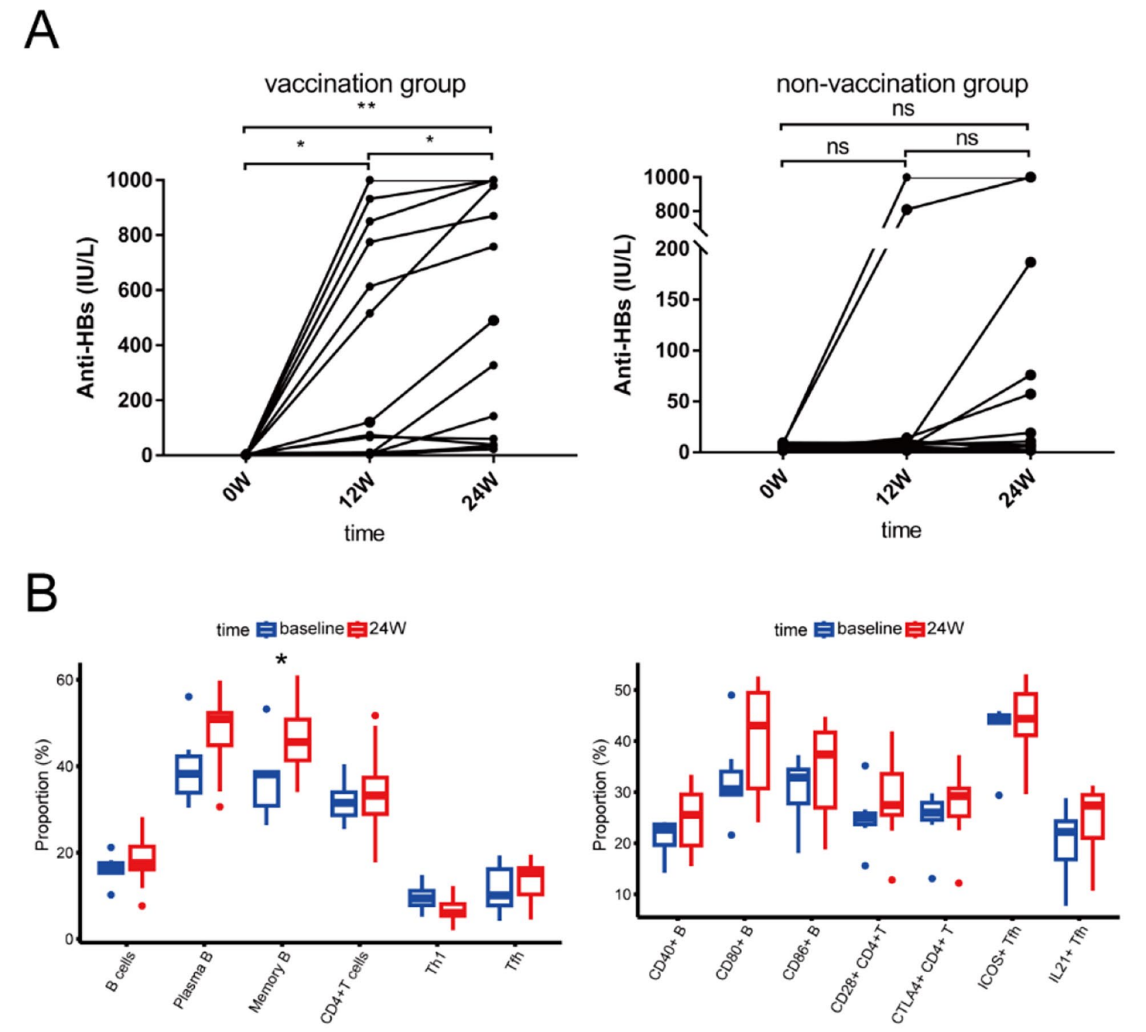
Changes of anti-HBs levels within groups and cell subsets in non-vaccine group. Comparison of anti-HBs levels at different time points in Vaccine group and non-vaccine group (**A**). Changes in proportion of cell subsets and the expression of surface cytokines in non-vaccine group (**B**). The statistical analysis of changes in anti-HBs was determined by Wilcoxon matched-pairs signed-rank test. The statistical analysis of changes in cell subsets was determined by independent sample t-test. *p < 0.05; **p < 0.005; ***p < 0.001

The anti-HBs-seropositivity rate at 24 weeks after vaccination was 100% in the vaccinated group, which was significantly higher than the 50% rate observed in the non-vaccinated group (Table 1). The anti-HBs levels were significantly higher at both 12 and 24 weeks in the vaccinated group than at the baseline (*p*-values of 0.006 and 0.001, respectively; Fig. 2A). In the non-vaccinated group, we observed increases, decreases, and non-significant changes in anti-HBs levels at 24 weeks when compared with baseline levels (Fig. 2A). Paired comparisons indicated no overall statistically significant differences (*p* > 0.05). With respect to anti-HBs levels between groups, the median 24-week anti-HBs level was significantly higher in the vaccinated group than in the non-vaccinated group (409.3 IUs/L vs. 8.97 IUs/L, respectively, *p* = 0.007). The proportions of individuals in the vaccinated group with anti-HBs levels of ≥ 100 IUs/L and ≥ 300 IUs/L were 64.3% and 57.1%, respectively, which were significantly higher than those in the non-vaccinated group (21.4% and 14.3%, respectively) (all *p*-values < 0.05; Table 1). Beyond common interferon-associated adverse reactions, the vaccinated group only exhibited slight redness, swelling, and pain at the vaccine-injection site, with no obvious abnormalities in the remaining patients.

### Changes in B cell subsets and surface-marker expression levels in the vaccinated group

**Fig. 3:**
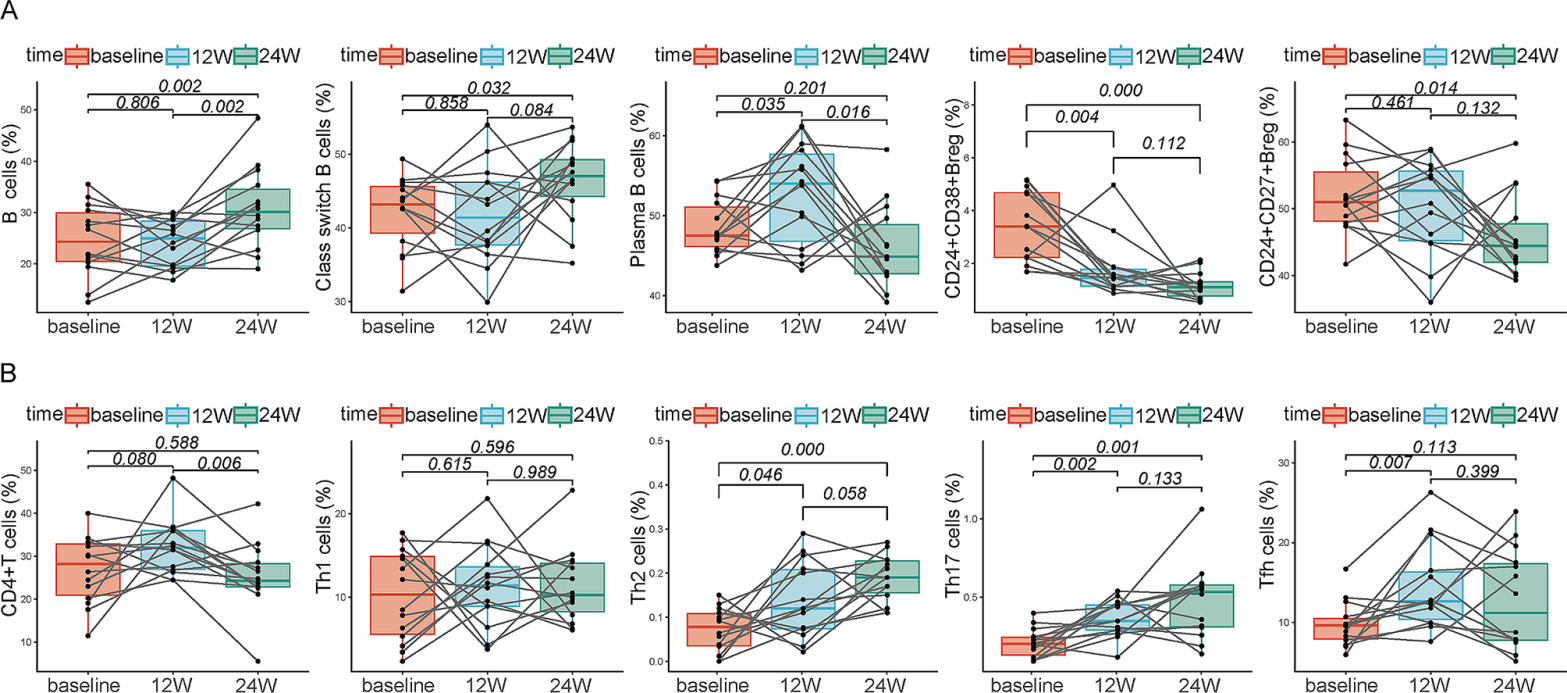
Changes of B cell and CD4^+^T cell subsets proportion of surface cytokines in vaccine group. Changes in the proportion of B cells, class switch B cells, plasma B cells, CD24^+^CD38^high^ Breg and CD24^+^CD27^+^ Breg in vaccine group at different time points (**A**). Changes in the proportion of CD4^+^T cells, Th1 cells, Th2 cells, Th17 cells and Tfh cells in vaccine group at different time points (**B**). The statistical analysis was determined by paired sample t-test

The proportion of total B cells was significantly higher in the vaccinated group at 24 weeks than at baseline (*p* = 0.002; Fig. 3A). The change in class-switched memory B cells was consistent with the overall change in B cells, which were significantly elevated from baseline at 24 weeks (all *p*-values < 0.05; Fig. 3A). The proportion of plasma cells also tended to be significantly higher and peaked at 12 weeks (*p* = 0.035; Fig. 3A). However, CD24^+^CD38^high^ regulatory B (Breg) and CD24^+^CD27^+^Breg cells were significantly lower at 24 weeks than at baseline (all *p*-values < 0.05; Fig. 3A).

**Fig. 4:**
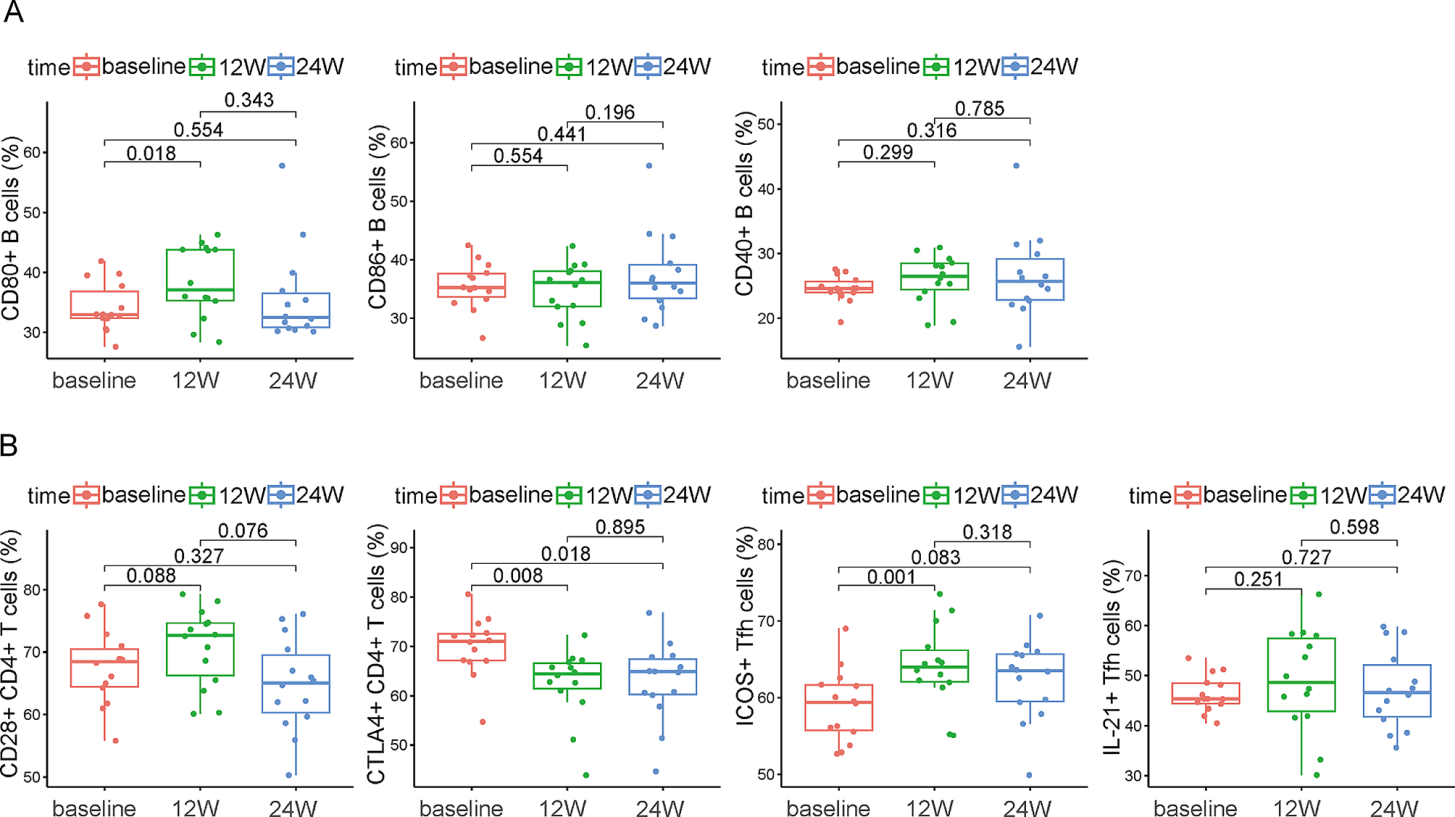
Changes of expression of surface cytokines in vaccine group. Changes in expression of CD80, CD86 and CD40 on surface of B cells in vaccine group at different time points (**A**). Changes in expression of CD40 and CTLA4 on surface of CD4^+^T cells, ICOS and IL-2 on surface of Tfh cells in vaccine group at different time points (**B**). The statistical analysis was determined by paired sample t-test

With respect to surface co-stimulatory molecule expression in B cell subsets, the proportions of CD80^+^B, CD86^+^B, and CD40^+^B cells all tended to increase (Fig. 4A). However, only the increase in CD80^+^B cells from the baseline to week 12 was statistically significant (*p* = 0.018; Fig. 4A).

### Changes in CD4^+^T cell subsets and surface-marker expression in the vaccinated group

The proportions of total CD4^+^T cells and Th1 cells tended to increase from baseline to 12 weeks, but the difference was not statistically significant (all *p*-values > 0.05; Fig. 3B). The proportions of Th2 cells and Th17 cells were significantly higher than baseline at 12 and 24 weeks (all *p*-values < 0.05; Fig. 3B). The proportion of follicular helper T cell (Tfh) cells was significantly higher at 24 weeks than at baseline (*p* = 0.007; Fig. 3B).

Regarding surface co-stimulatory molecule expression in CD4^+^T cell subsets, the proportions of CD28^+^CD4^+^T, ICOS^+^Tfh, and IL-21^+^Tfh cells all increased from baseline to 12 weeks, but only the difference in ICOS^+^Tfh cells was statistically significant (*p* = 0.001; Fig. 4B). CTLA4^+^CD4^+^T cells tended to decrease and were significantly lower than baseline at both 12 and 24 weeks (*p*-values of 0.008 and 0.018, respectively; Fig. 4B).

### Changes in cell-subset proportions and surface-marker expression in the non-vaccinated group

Among the changes that occurred in different cell subsets in the non-vaccinated group from baseline to 24 weeks, the proportions of total B, memory B, plasma, CD4^+^T, and Tfh cells all tended to increase, but only the difference in memory B cells was statistically significant (*p* < 0.05; Fig. 2B). With respect to the expression of cell-surface co-stimulatory/inhibitory molecules, the proportions of CD40^+^B, CD80^+^B, CD28^+^CD4^+^T, CTLA4^+^CD4^+^T, ICOS^+^Tfh, and IL-21^+^Tfh cells tended to increase at 24 weeks, but these differences were not statistically significant from those at the baseline (all *p*-values > 0.05; Fig. 2B).

### Changes in plasma cytokines and immunoglobulins

**Fig. 5:**
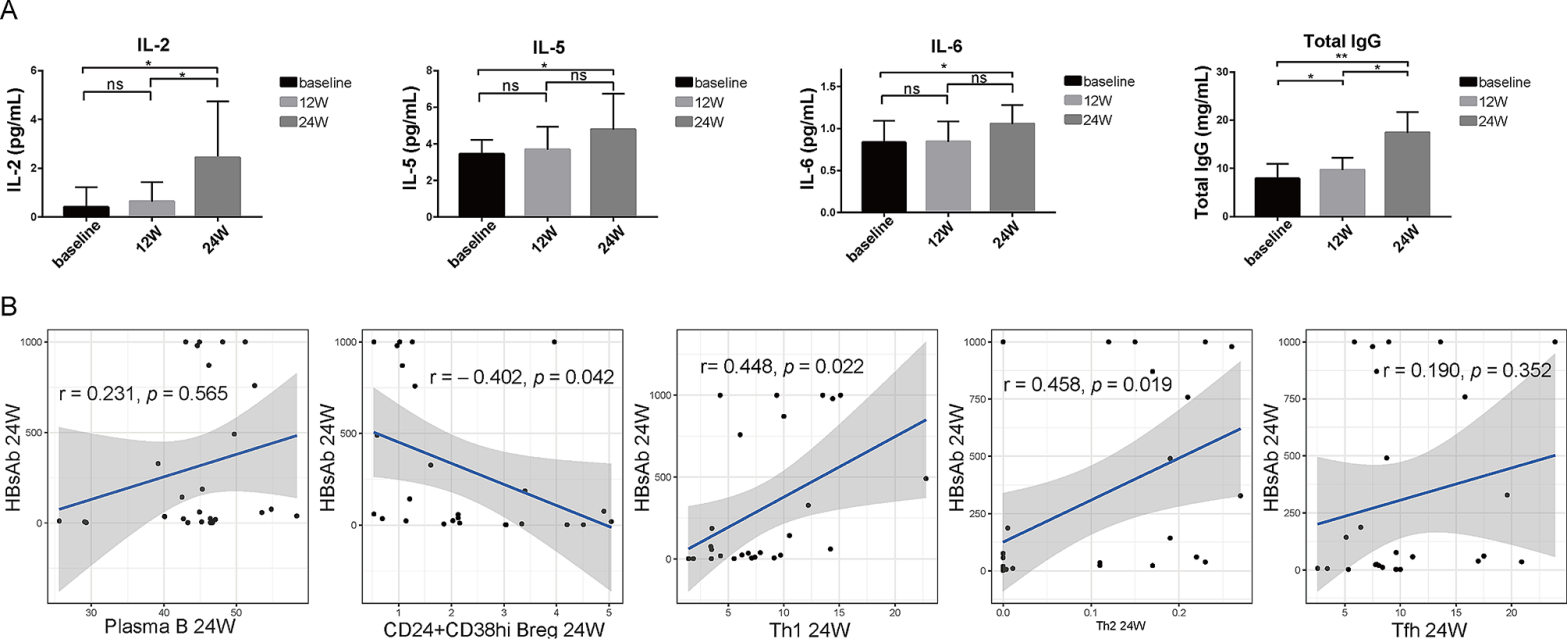
Changes of cytokines and immunoglobulins in serum, correlation between anti-HBs and proportion of cell subsets. Changes of IL-2, IL-5, IL-6 and total IgG in serum of Vaccine group at different time points (**A**). Correlations between anti-HBs and the proportion of plasma B cells, CD24^+^CD38^high^ Breg, Th1 cells Th2 cells and Tfh cells at week 24 (**B**). The statistical analysis of changes in cytokines and immunoglobulins was determined by Wilcoxon matched-pairs signed-rank test. The level of anti-HBs and the proportion of cell subsets were evaluated by spearman correlation. *p < 0.05; **p < 0.005; ***p < 0.001

Changes in the plasma levels of cytokines such as IFN-γ and IL-2 were determined at different time points in both groups. In the vaccinated group, IL-2, IL-5, and IL-6 levels were significantly higher at 24 weeks than at baseline (all *p*-values < 0.05; Fig. 5A). The changes in the remaining cytokines were not significant (all *p*-values > 0.05; Supplementary Fig. 1). In the non-vaccinated group, no significant changes were observed in these cytokines at 24 weeks compared to baseline levels (all *p*-values > 0.05; Supplementary Fig. 2).

With respect to changes in plasma Ig, total IgG tended to be significantly higher than baseline at both 12 and 24 weeks in the vaccinated group (*p*-values of 0.016 and 0.004, respectively; Fig. 5A). In the non-vaccinated group, IgG levels did not differ significantly at 24 weeks compared to that at baseline (all *p*-values > 0.05; Supplementary Fig. 2).

### Correlations between anti-HBs and cell-subset proportions

We analyzed correlations between anti-HBs and the relative proportions of cell subsets in all 28 patients. The anti-HBs titer at 24 weeks correlated positively with the proportions of plasma, Th1, Th2, and Tfh cells in the peripheral blood (Fig. 5B). The correlation between Th1 and Th2 cells was statistically significant (r-values of 0.488 and 0.458, respectively; *p*-values of 0.022 and 0.019, respectively; Fig. 5B). The anti-HBs titer exhibited a significant negative correlation with the proportion of CD24^+^CD38^high^Breg cells (*r* = -0.402, *p* = 0.042).

## Discussion

With the optimization of hepatitis B antiviral-treatment strategies, the number of patients achieving HBsAg seroclearance has increased [11, 12]. In turn, reducing HBsAg recurrence has become a major clinical issue of concern. Several clinical studies have consistently demonstrated that anti-HBs seroconversion and high antibody levels are strongly associated with HBsAg recurrence seroclearance [6, 8, 13]. In a prospective study involving 238 patients who achieved HBsAg seroclearance following Peg-IFNα treatment with a median follow-up period of 160 weeks, we observed a cumulative HBsAg recurrence rate of 9.66% was reported. The recurrence rate was significantly lower for those with ≥ 100 IUs anti-HBs/L than for those with < 100 IUs anti-HBs/L (4.3% vs. 21.1%, *p* < 0.001) [7]. A meta-analysis of 43,924 patients further showed that anti-HBs seroconversion was a protective factor against recurrence after HBsAg seroclearance [14]. Additionally, the incidence of HCC was much lower in patients who achieved HBsAg seroclearance than in those who did not (RR = 0.41, *p* < 0.001). Recent findings also suggest that hepatitis B vaccination may increase anti-HBs levels and thereby help reduce the recurrence rate [9, 10, 15]. The results of a recent retrospective study suggested that hepatitis B vaccination can significantly increase anti-HBs-positive conversion and antibody levels. Vaccination emerged as the major influencing factor enabling anti-HBs levels to reach 100 IUs/L (OR = 4.396, *p* < 0.001) [16]. Furthermore, Jiang et al. found that individuals who achieving HBsAg seroclearance after IFNα-based therapy had significantly different HBsAg-recurrence rates based on their vaccination status: 7.7% in vaccinated individuals with ≥ 100 IUs anti-HBs/L, 58.5% in vaccinated with < 100 IUs anti-HBs/L, and 31.9% in unvaccinated individuals (*p* < 0.05 in each case) [15]. Despite the increasing number of patients achieving HBsAg seroclearance due to optimized hepatits B antiviral-treatment strategies, there is limited literature on the immune function changes following hepatitis B vaccination in these patients. To address this gap, we formed a cohort of patients who achieved HBsAg seroclearance after Peg-IFNα treatment. We then categorized them based on whether they received hepatitis B vaccination after HBsAg seroclearance, and investigated the changes in their antibody levels and immune functions.

First, we analyzed differences in antibody production between the groups with or without hepatitis B vaccination. In terms of intra-group comparisons, Anti-HBs levels increased significantly from baseline at both 12 and 24 weeks in the vaccinated group (all *p*-values < 0.05), whereas they did not change significantly from baseline to either time point in the non-vaccinated group (all *p*-values > 0.05). In terms of inter-group comparisons, the anti-HBs-seroconversion rate reached 100% in the vaccinated group at 24 weeks but only 50% in the non-vaccinated group (*p* = 0.006. The proportions of individuals showing anti-HBs levels of ≥ 100 IUs/L or ≥ 300 IUs/L at 24 weeks in the vaccinated group were significantly higher than those in the non-vaccinated group (all *p*-values < 0.05). The results of similar studies showed that the anti-HBs-seroconversion rates post-hepatitis B vaccination ranged from 81.8 to 100% in individuals with HBsAg seroclearance after IFNα-based therapy, compared to 0–36.4% in unvaccinated individuals [9, 10, 15]. Patients with CHB who were treated with Peg-IFNα for 24 weeks showed significant increases in memory B and plasma cells, relative to baseline levels [17]. In contrast, untreated patients with CHB had difficulty producing anti-HBs even when administered multiple hepatitis B-vaccine doses, which may relate to severe B cell dysfunction in patients with CHB [18, 19]. In vitro data showed that B cells from patients with HBsAg seroclearance reversed B cell dysfunction and facilitated anti-HBs production through co-stimulation with HBsAg and other cytokines [20]. Therefore, in this study, we selected patients with HBsAg seroclearance following Peg-IFNα treatment for hepatitis B vaccine. Our results showed that the rate of anti-HBs seroconversion and antibody levels significantly increased, indicating that vaccination is effective clinically for reducing recurrence in patients with HBsAg seroclearance. Furthermore, no new adverse effects were observed with increased doses of the hepatitis B vaccine.

To investigate the variability of immunity in individuals with HBsAg seroclearance with or without hepatitis B vaccination, we examined lymphocytes from peripheral blood samples. Compared to baseline levels, the vaccinated group had a significantly higher proportion of plasma cells at 12 weeks and higher proportions of total B cells and class-switched memory B cells at 24 weeks (all *p*-values < 0.05; Fig. 3A). In the vaccinated group, plasma anti-HBs titers and total IgG concentrations increased significantly from baseline to 24 weeks, suggesting that HBsAg seroclearance followed by vaccination resulted in significantly enhanced humoral immune function. Huang et al. also showed that total B cells and plasma cells were significantly higher in a group with ≥ 100 U/L anti-HBs than in a group with < 100 U/L anti-HBs, among individuals demonstrating HBsAg seroclearance following Peg-IFNα treatment [8]. Other findings showed that the proportion of class-switched memory B cells increased after stimulation with exogenous antigens, such as vaccines. These cells could further differentiate into plasma cells to produce antibodies [21–23]. In this study, we also found significantly higher levels of class-switched memory B cells after vaccination. In addition to an elevated percentage of B cells, we also found significant increases in co-stimulatory molecules associated with cell activation. The results of this study showed a significantly higher proportion of CD80^+^B cells at 12 weeks than at baseline in the vaccinated group (*p* = 0.018). The expression of CD80, a co-stimulatory B cell-surface molecule, reflects the antigen-presentation function of B cells. Studies on CD80 expression in the context of hepatitis B vaccination have not been reported, although similar studies have been conducted for HIV-infected individuals. Powell et al. observed that among HIV-infected patients who received an influenza vaccine, B cell-surface CD80 expression was significantly higher in those who responded to the vaccine (antibody titers increased by over 4-fold after 7 days) but not in non-responders [24]. Tfh cells are required for T cell-dependent B cell maturation and play key roles in memory B and plasma cell differentiation. As shown in Fig. 3B, the proportion of Tfh cells significantly increased from baseline to 12 weeks, which is consistent with the changes observed in plasma cells (*p* = 0.007). ICOS, an important receptor on the surface of Tfh cells, promoted their proliferation upon binding to its ligand on the surface of B cells [25]. In our study, we observed that the proportion of ICOS^+^Tfh cells increased significantly from baseline to 12 weeks in the vaccinated group (*p* = 0.001). However, in the non-vaccinated group, the changes in Tfh and ICOS^+^Tfh cells were not significant. Previous reports on changes in Tfh cells and ICOS expression after hepatitis B vaccination in individuals with HBsAg seroclearance focused on changes during the Peg-IFNα-treatment period. Zhang et al. reported that elevated Tfh cells after 48 weeks of Peg-IFNα treatment correlated significantly with decreased HBsAg levels [26]. Liu et al. observed a gradual increase in the proportion of ICOS^+^Tfh cells in individuals with HBsAg seroclearance during Peg-IFNα treatment, peaking at week 48, whereas the difference in individuals without HBsAg seroclearance was not significant [27]. Previous data have also suggested that several vaccines, including a hepatitis B vaccine, cause elevated levels of Tfh cells in healthy adults [28–30]. In addition, the proportion of ICOS^+^Tfh cells was lower in healthy adults who did not receive hepatitis B vaccination or had a weak response to it, suggesting that Tfh cells and ICOS are involved in humoral immunity and promote the production of anti-HBs antibodies [30]. However, we did not measure HBsAg-specific B cell and Tfh cell ratios. To fully understand the exact mechanism involved, further investigation is need.

Among B cell subsets, Breg cells play a negative regulatory role. For example, IL-10 secretion by Breg cells inhibits B-cell activation and leads to reduced antibody production [31, 32]. The present findings demonstrated that the abundances of both types of Breg cells in the vaccinated group decreased significantly compared to baseline levels during treatment. Anti-HBs levels showed a significant negatively correlated with the proportion of CD24^+^CD38^high^Breg cells (all *p*-values < 0.05; Fig. 3A). The differences in the number of Breg cells were not significant in the non-vaccinated group. Previous reports revealed that, overall, Breg cells increased significantly during the early stage of Peg-IFNα treatment (12–24 weeks) [17, 33]. However, Fu et al. showed that with extended Peg-IFNα treatment, the proportion of Breg cells gradually decreased, which correlated significantly with a higher HBeAg-seroconversion rate and higher IgG secretion [33]. Those results are consistent with our present findings. In contrast, Breg cells were significantly elevated in untreated patients with CHB, which represent the primary source of elevated IL-10 production [34]. No reports have described changes in Breg cell abundances in individuals with HBsAg seroclearance after Peg-IFNα treatment and hepatitis B vaccination. However, changes in Breg cells following hepatitis B vaccination have been better studied in healthy adults. It is generally believed that lower numbers of Breg cells are associated with higher anti-HBs-antibody production. Previous findings showed that among healthy adults vaccinated against hepatitis B, the proportions of CD24^+^CD27^+^Breg and CD24^+^CD38^high^ Breg cells were significantly lower in the anti-HBs-positive group than in the anti-HBs-negative group [35]. In this study, these two types of Breg cells were significantly less abundant in the vaccinated group during treatment. In another study, the high-anti-HBs group showed fewer CD24^+^CD38^high^Breg cells and reduced IL-10 production [36]. Therefore, the present findings suggest that the concomitant use of Peg-IFNα and hepatitis B vaccination in individuals with HBsAg seroclearance may improve humoral immune function.

Th cells and their cytokines play regulatory roles during multiple stages of humoral immunity, including B cell development, differentiation, and antibody production [37]. We observed a significant positive correlation between anti-HBs antibodies and the proportion of Th2 cells at week 24 in the overall patient cohort (*r* = 0.456, *p* = 0.019). Among patients in the vaccinated group, Th2/Th17 cells were significantly higher at 12 and 24 weeks than at baseline (all *p*-values < 0.05; Fig. 3B). In addition, the IL-2, IL-5, and IL-6 concentrations were significantly higher in the vaccinated group at 24 weeks than at baseline (all *p*-values < 0.05). However, no statistically significant changes were observed in the non-vaccinated group. Thus, we hypothesize that Th cells may contribute to anti-HBs production. While few reports have described the correlation between Th cells and HBsAg seroclearance, Islam et al. found that Peg-IFNα drove Th1/Th17 cell differentiation, and increased Th1/Th17 cells were associated with HBsAg seroclearance [38]. In addition, Doedée et al. reported that highly active Th2 cells were associated with higher anti-HBs-antibody titers in healthy adults who were vaccinated against hepatitis B [39].

In conclusion, the present findings suggest that hepatitis B vaccination in individuals with HBsAg seroclearance significantly enhances the anti-HBs-seroconversion rate and increases antibody levels. Therefore, we believe that concomitant hepatitis B vaccination to increase anti-HBs levels during the late stage of Peg-IFNα treatment may be an effective measure for preventing recurrence. Our results also revealed significant increases in plasma, Tfh, CD80^+^B, and ICOS^+^Tfh cells and a significant decrease in Breg cells during treatment. These results suggest that treatment with Peg-IFNα plus hepatitis B vaccination strongly promotes the restoration of humoral immunity in CHB-infected patients. However, due to the small sample size analyzed in this study and the absence of decriptions for HBsAg-specific B and T cells, our conclusions require further validation in future studies.

### Electronic supplementary material

Below is the link to the electronic supplementary material.

Supplementary Material 1

Supplementary Material 2

## Data Availability

The original contributions presented in the study are included in the article/Supplementary Material.

## Abbreviations

ALT: alanine aminotransferase
anti-HBs: antibodies
CHB: chronic hepatitis B
Tfh: follicular helper T cell
HBsAg: hepatitis B surface antigen
HBV: Hepatitis B virus
HCC: hepatocellular carcinoma
Ig: immunoglobulin
IL: interleukins
Peg-IFNα: Pegylated-interferonα
PBMC: Peripheral blood mononuclear cell
PBS buffer: phosphate buffered saline solution
TNF: tumor necrosis factor

## References

[1] Liaw YF (2009). Antiviral therapy of chronic hepatitis B: opportunities and challenges in Asia. J Hepatol.

[2] Moucari R, Korevaar A, Lada O, Martinot-Peignoux M, Boyer N, Mackiewicz V, Dauvergne A, Cardoso AC, Asselah T, Nicolas-Chanoine MH (2009). High rates of HBsAg seroconversion in HBeAg-positive chronic hepatitis B patients responding to interferon: a long-term follow-up study. J Hepatol.

[3] Kim GA, Lim YS, An J, Lee D, Shim JH, Kim KM, Lee HC, Chung YH, Lee YS, Suh DJ (2014). HBsAg seroclearance after nucleoside analogue therapy in patients with chronic hepatitis B: clinical outcomes and durability. Gut.

[4] Terrault NA, Lok ASF, McMahon BJ, Chang KM, Hwang JP, Jonas MM, Brown RS, Bzowej NH, Wong JB (2018). Update on prevention, diagnosis, and treatment of chronic hepatitis B: AASLD 2018 hepatitis B guidance. Hepatology.

[5] EASL. 2017 Clinical Practice Guidelines on the management of hepatitis B virus infection. J Hepatol 2017, 67:370–398. 10.1016/j.jhep.2017.03.021 28427875

[6] Li MH, Yi W, Zhang L, Lu Y, Lu HH, Shen G, Wu SL, Hao HX, Gao YJ, Chang M (2019). Predictors of sustained functional cure in hepatitis B envelope antigen-negative patients achieving hepatitis B surface antigen seroclearance with interferon-alpha-based therapy. J Viral Hepat.

[7] Wu Y, Liu Y, Lu J, Cao Z, Jin Y, Ma L, Geng N, Ren S, Zheng Y, Shen C, Chen X (2020). Durability of Interferon-induced Hepatitis B Surface Antigen Seroclearance. Clin Gastroenterol Hepatol.

[8] Huang D, Wu D, Wang P, Wang Y, Yuan W, Hu D, Hu J, Wang Y, Tao R, Xiao F (2022). End-of-treatment HBcrAg and HBsAb levels identify durable functional cure after Peg-IFN-based therapy in patients with CHB. J Hepatol.

[9] Zeng QL, Yu ZJ, Shang J, Xu GH, Sun CY, Liu N, Li CX, Lv J, Liu YM, Liang HX (2020). Short-term Peginterferon-Induced High Functional Cure Rate in Inactive Chronic Hepatitis B Virus Carriers with Low Surface Antigen levels. Open Forum Infect Dis.

[10] Wu F, Wang Y, Cui D, Tian Y, Lu R, Liu C, Li M, Li Y, Gao N, Jiang Z et al. Short-term Peg-IFN α-2b Re-treatment Induced a high functional cure rate in patients with HBsAg recurrence after stopping Peg-IFN α-Based regimens. J Clin Med 2023, 12. 10.3390/jcm12010361 PMC9821570 36615161

[11] Cao Z, Liu Y, Ma L, Lu J, Jin Y, Ren S, He Z, Shen C, Chen X (2017). A potent hepatitis B surface antigen response in subjects with inactive hepatitis B surface antigen carrier treated with pegylated-interferon alpha. Hepatology.

[12] Wu YL, Shen CL, Chen XY (2019). Antiviral treatment for chronic hepatitis B: safety, effectiveness, and prognosis. World J Clin Cases.

[13] Pan CQ, Li MH, Yi W, Zhang L, Lu Y, Hao HX, Wan G, Cao WH, Wang XY, Ran CP (2021). Outcome of Chinese patients with hepatitis B at 96 weeks after functional cure with IFN versus combination regimens. Liver Int.

[14] Song A, Wang X, Lu J, Jin Y, Ma L, Hu Z, Zheng Y, Shen C, Chen X (2021). Durability of hepatitis B surface antigen seroclearance and subsequent risk for hepatocellular carcinoma: a meta-analysis. J Viral Hepat.

[15] Jiang S, Cai M, Zhang Z, Qian C, Wang J, Li Z, Guo Q, Zhou H, Xin H, Cai W et al. The potential effect of HBV vaccination on off-treatment HBsAg reversion after interferon-induced HBsAg clearance. Hum Vaccin Immunother 2023:2161254. 10.1080/21645515.2022.2161254 PMC9980474 36683193

[16] Liu Y, Ren S, Ma L, Lin X, Li H, Lu J et al. Clinical study of hepatitis B vaccine in achieving hepatitis B surface antibody seroconversion in patients with functional cure. Braz J Infect Dis, 2023:103703. 10.1016/j.bjid.2023.103703 PMC10698567 38036020

[17] Wu ZQ, Tan L, Gan WQ, Mo ZS, Chen DB, Wang PP, Zhao QY, Xie DY, Gao ZL (2021). The relationship between the clearance of HBsAg and the remodeling of B cell subsets in CHB patients treated with Peg-IFN-α. Ann Transl Med.

[18] Xu X, Shang Q, Chen X, Nie W, Zou Z, Huang A, Meng M, Jin L, Xu R, Zhang JY (2015). Reversal of B-cell hyperactivation and functional impairment is associated with HBsAg seroconversion in chronic hepatitis B patients. Cell Mol Immunol.

[19] Bertoletti A, Ferrari C (2016). Adaptive immunity in HBV infection. J Hepatol.

[20] Salimzadeh L, Le Bert N, Dutertre CA, Gill US, Newell EW, Frey C, Hung M, Novikov N, Fletcher S, Kennedy PT, Bertoletti A (2018). PD-1 blockade partially recovers dysfunctional virus-specific B cells in chronic hepatitis B infection. J Clin Invest.

[21] Wu YC, Kipling D, Leong HS, Martin V, Ademokun AA, Dunn-Walters DK (2010). High-throughput immunoglobulin repertoire analysis distinguishes between human IgM memory and switched memory B-cell populations. Blood.

[22] Vásquez C, Franco MA, Angel J (2015). Rapid Proliferation and differentiation of a subset of circulating IgM Memory B cells to a CpG/Cytokine stimulus in Vitro. PLoS ONE.

[23] McHeyzer-Williams LJ, Milpied PJ, Okitsu SL, McHeyzer-Williams MG (2015). Class-switched memory B cells remodel BCRs within secondary germinal centers. Nat Immunol.

[24] Rinaldi S, Pallikkuth S, George VK, de Armas LR, Pahwa R, Sanchez CM, Pallin MF, Pan L, Cotugno N, Dickinson G (2017). Paradoxical aging in HIV: immune senescence of B cells is most prominent in young age. Aging.

[25] Weinstein JS, Bertino SA, Hernandez SG, Poholek AC, Teplitzky TB, Nowyhed HN, Craft J (2014). B cells in T follicular helper cell development and function: separable roles in delivery of ICOS ligand and antigen. J Immunol.

[26] Zhang L, Li H, Ren H, Hu P (2018). Circulating PD-1(hi)CXCR5(+)CD4(+) T cells are associated with a decrease in hepatitis B surface antigen levels in patients with chronic hepatitis B who are receiving peginterferon-α therapy. Mol Immunol.

[27] Liu Y, Hu X, Hu X, Yu L, Ji H, Li W, Cai Y, Cheng G, Jiang Y (2022). T follicular helper cells improve the response of patients with chronic hepatitis B to interferon by promoting HBsAb production. J Gastroenterol.

[28] Bentebibel SE, Khurana S, Schmitt N, Kurup P, Mueller C, Obermoser G, Palucka AK, Albrecht RA, Garcia-Sastre A, Golding H, Ueno H (2016). ICOS(+)PD-1(+)CXCR3(+) T follicular helper cells contribute to the generation of high-avidity antibodies following influenza vaccination. Sci Rep.

[29] Heit A, Schmitz F, Gerdts S, Flach B, Moore MS, Perkins JA, Robins HS, Aderem A, Spearman P, Tomaras GD (2017). Vaccination establishes clonal relatives of germinal center T cells in the blood of humans. J Exp Med.

[30] Xing M, Feng Y, Yao J, Lv H, Chen Y, He H, Wang Z, Hu C, Lou X (2020). Induction of peripheral blood T follicular helper cells expressing ICOS correlates with antibody response to hepatitis B vaccination. J Med Virol.

[31] Chekol Abebe E, Asmamaw Dejenie T, Mengie Ayele T, Dagnew Baye N, Agegnehu Teshome A, Tilahun Muche Z (2021). The Role of Regulatory B Cells in Health and diseases: a systemic review. J Inflamm Res.

[32] Sanaei MJ, Nahid-Samiei M, Abadi MSS, Arjmand MH, Ferns GA, Bashash D, Rahimian G, Bagheri N (2021). New insights into regulatory B cells biology in viral, bacterial, and parasitic infections. Infect Genet Evol.

[33] Fu B, Wang D, Shen X, Guo C, Liu Y, Ye Y, Sun R, Li J, Tian Z, Wei H (2020). Immunomodulation Induced during Interferon-α therapy impairs the Anti-HBV Immune Response through CD24(+)CD38(hi) B cells. Front Immunol.

[34] Gong Y, Zhao C, Zhao P, Wang M, Zhou G, Han F, Cui Y, Qian J, Zhang H, Xiong H (2015). Role of IL-10-Producing Regulatory B cells in Chronic Hepatitis B Virus infection. Dig Dis Sci.

[35] Körber N, Pohl L, Weinberger B, Grubeck-Loebenstein B, Wawer A, Knolle PA, Roggendorf H, Protzer U, Bauer T (2021). Hepatitis B Vaccine Non-responders Show Higher frequencies of CD24(high)CD38(high) Regulatory B cells and lower levels of IL-10 expression compared to responders. Front Immunol.

[36] Bolther M, Andersen KLD, Tolstrup M, Visvanathan K, Woolley I, Skinner N, Millen R, Warner N, Østergaard L, Jensen-Fangel S (2018). Levels of regulatory B cells do not predict serological responses to hepatitis B vaccine. Hum Vaccin Immunother.

[37] McHeyzer-Williams LJ, McHeyzer-Williams MG (2005). Antigen-specific memory B cell development. Annu Rev Immunol.

[38] Islam M, Kumar K, Sevak JK, Jindal A, Vyas AK, Ramakrishna G, Kottilil S, Sharma MK, Sarin SK, Trehanpati N. Immune drivers of HBsAg loss in HBeAg-negative CHB patients after stopping nucleotide analog and administration of Peg-IFN. Hepatol Commun 2023, 7. 10.1097/HC9.0000000000000098 PMC10145869 37102765

[39] Doedée AM, Kannegieter N, Öztürk K, van Loveren H, Janssen R, Buisman AM (2016). Higher numbers of memory B-cells and Th2-cytokine skewing in high responders to hepatitis B vaccination. Vaccine.

