# Supplementary material for: Peg-IFNα combined with hepatitis B vaccination contributes to HBsAg seroconversion and improved immune function
Source: Virol J. 2024 Mar 30;21:77. doi: 10.1186/s12985-024-02344-8 (PMC10981809; doi:10.1186/s12985-024-02344-8)
Supplement: Supplementary file 1 — Supplementary Material 1 [file 12985_2024_2344_MOESM1_ESM.docx]

**Supplementary Tables**

**Supplementary Table 1 Mass cytometry Panel Design**

| Antigen | Symbol and Mass | Antibody clone | Source |
| --- | --- | --- | --- |
| CXCR3 | 141 | 49801 | R＆D |
| CD40 | 142 | 5C3 | Fluidigm |
| CCR6 | 143 | 53103 | R＆D |
| CD38 | 144 | HIT2 | Fluidigm |
| CD4 | 145 | RPA-T4 | Fluidigm |
| CD20 | 147 | 2H7 | Fluidigm |
| ICOS | 148 | C398.4A | Fluidigm |
| CD3 | 149 | 2A3 | Biolegned |
| CD45 | 152 | HI30 | Biolegned |
| CD86 | 153 | IT2.2 | Biolegned |
| CD25 | 154 | 24212 | R＆D |
| CD45 | 155 | HI30 | Biolegned |
| CD45 | 156 | HI30 | Biolegned |
| IL-21 | 158 | EPR22618-28 | abcam |
| PD-1 | 159 | RMP1-30 | Fluidigm |
| CD28 | 160 | CD28.2 | Fluidigm |
| CTLA4 | 161 | 14D3 | Fluidigm |
| CD80 | 163 | 37711 | R＆D |
| CD19 | 165 | HIB19 | Fluidigm |
| IgD | 166 | IA6-2 | R＆D |
| CCR7 | 167 | G043H7 | Fluidigm |
| CD24 | 169 | ML5 | Fluidigm |
| CD27 | 170 | [O323](https://www.biolegend.com/en-us/search-results?Clone=O323) | Biolegned |
| CXCR5 | 171 | RF8B2 | Fluidigm |
| CD45 | 172 | HI30 | Biolegned |

**Supplementary Table 2** Comparison of baseline cytokine and immunoglobulin levels

|  | Vaccine group (n=14) | Non-vaccine group (n=14) | p value |
| --- | --- | --- | --- |
| Median IFN-gamma (pg/mL) | 0.76 | 0.68 | 0.537 |
| Median IL-2 (pg/mL) | 0.41 | 0.34 | 0.431 |
| Median IL-4 (pg/mL) | 1.63 | 1.04 | 0.338 |
| Median IL-5 (pg/mL) | 3.44 | 2.39 | 0.206 |
| Median IL-6 (pg/mL) | 0.44 | 0.59 | 0.160 |
| Median IL-10 (pg/mL) | 5.90 | 6.41 | 0.758 |
| Median IL-12 (pg/mL) | 6.01 | 4.40 | 0.217 |
| Median IL-17A (pg/mL) | 1.45 | 1.01 | 0.355 |
| Median IL-22 (pg/mL) | 1.78 | 2.71 | 0.356 |
| Median TNF-beta (pg/mL) | 0.68 | 0.38 | 0.515 |
| Median IgM (mg/mL) | 0.77 | 0.59 | 0.632 |
| Median IgG (mg/mL) | 7.87 | 9.07 | 0.195 |
| Median IgA (mg/mL) | 1.60 | 1.47 | 0.608 |
