# Supplementary material for: Peg-IFNα combined with hepatitis B vaccination contributes to HBsAg seroconversion and improved immune function
Source: Virol J. 2024 Mar 30;21:77. doi: 10.1186/s12985-024-02344-8 (PMC10981809; doi:10.1186/s12985-024-02344-8)

**Supplementary Figures**

**Supplementary Fig. 1** The changes of cytokines in serum of Vaccine group


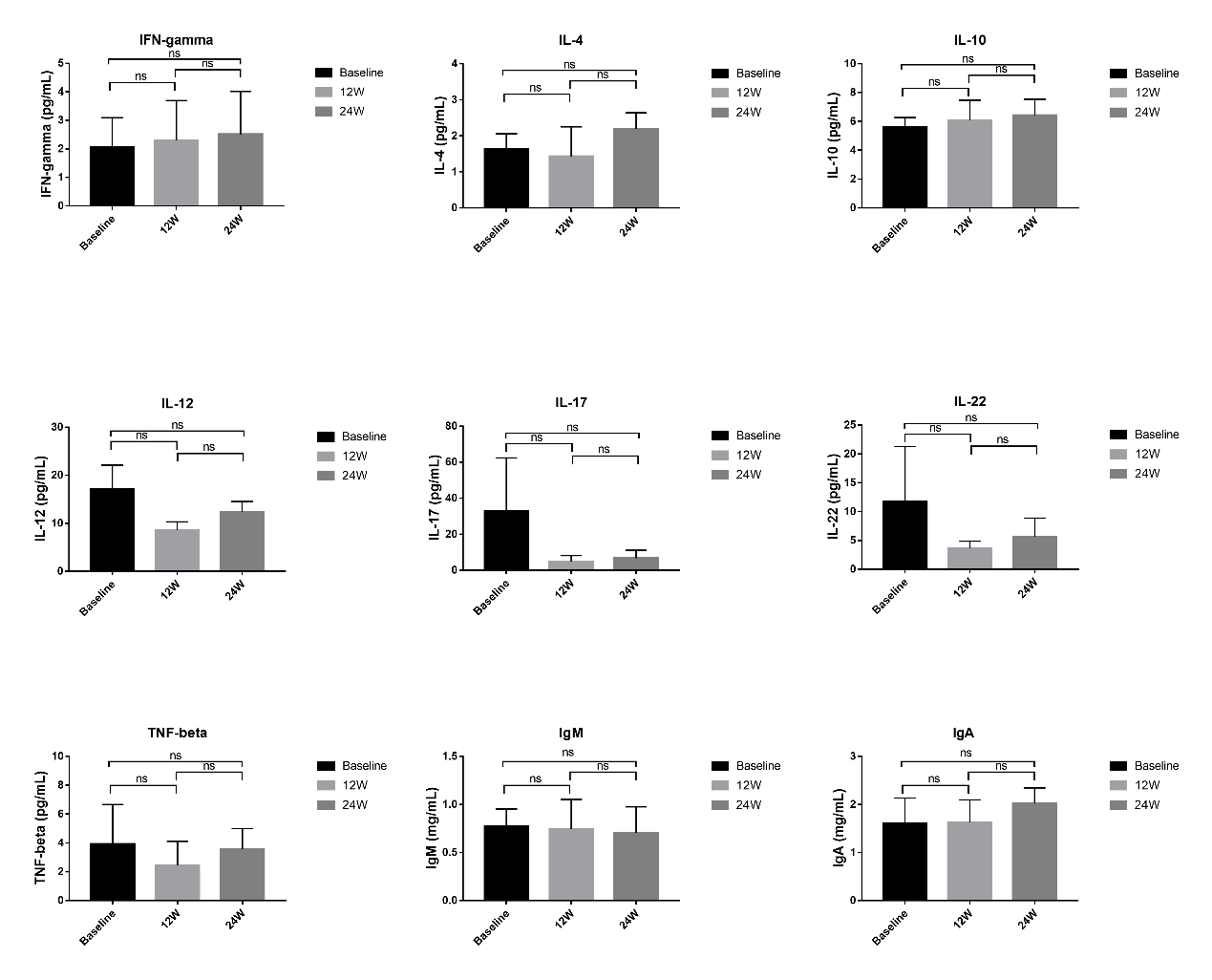


**Supplementary Fig. 2** The changes of cytokines and immunoglobulins in serum of Non-vaccine group


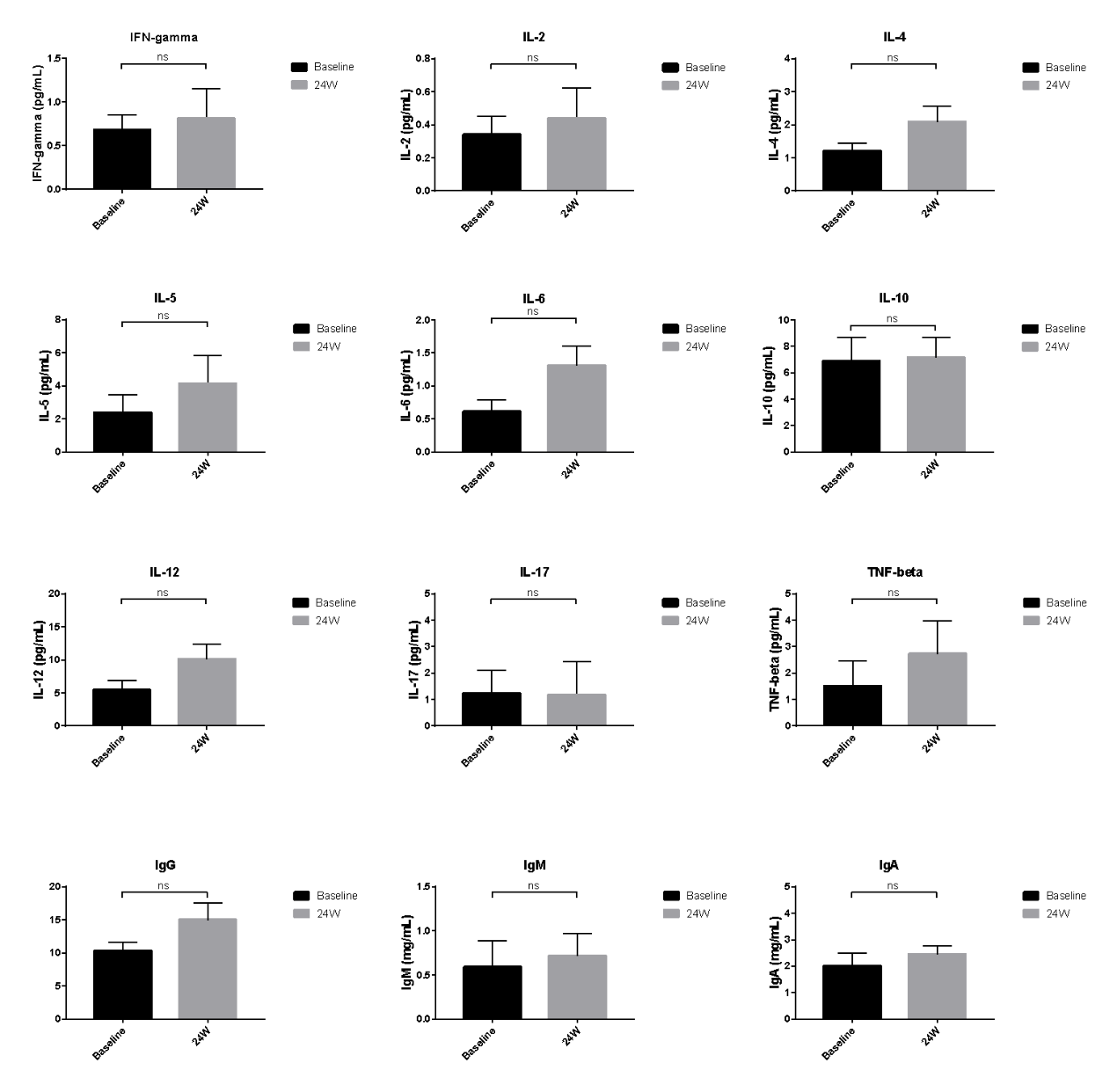

Supplement: Supplementary file 2 — Supplementary Material 2 [file 12985_2024_2344_MOESM2_ESM.docx]
